# Prevalence of *Cryptosporidium* in small ruminants from Veracruz, Mexico

**DOI:** 10.1186/s12917-016-0638-3

**Published:** 2016-01-19

**Authors:** Dora Romero-Salas, Cosme Alvarado-Esquivel, Anabel Cruz-Romero, Mariel Aguilar-Domínguez, Nelly Ibarra-Priego, José O. Merino-Charrez, Adalberto A. Pérez de León, Jesús Hernández-Tinoco

**Affiliations:** Laboratorio de Parasitología. UD PZTM. Facultad de Medicina Veterinaria y Zootecnia, Universidad Veracruzana, Veracruz, Mexico; Laboratorio de Investigación Biomédica. Facultad de Medicina y Nutrición, Juárez University of Durango State, Avenida Universidad S/N, 34000 Durango, Mexico; Facultad de Medicina Veterinaria y Zootecnia, Universidad Autónoma de Tamaulipas, Cd. Victoria, Tamaulipas, Mexico; USDA-ARS Knipling-Bushland U.S. Livestock Insects Research Laboratory, and Veterinary Pest Genomics Center, Kerrville, TX USA; Institute for Scientific Research “Dr. Roberto Rivera Damm”, Juárez University of Durango State, Durango, Mexico

**Keywords:** *Cryptosporidium*, Infection, Zonoosis, Prevalence, Small ruminants, Sheep, Goats, Veracruz State, Mexico

## Abstract

**Background:**

Cryptosporidiosis is a zoonotic disease caused by the protozoan parasite *Cryptosporidium* spp. that can affect domestic animal and human populations. In newborn ruminants, cryptosporidiosis is characterized by outbreaks of diarrhea, which can result in high morbidity and economic impact. The aim of the present study was to determine the prevalence of *Cryptosporidium* spp. in small ruminants from the Perote municipality in Veracruz State, Mexico. One hundred and sixty small ruminants (80 sheep and 80 goats) from eight farms located in four towns of the Perote municipality were examined following a cross-sectional study design. Stool samples were analyzed by a modification of the Faust centrifugation method, and the presence of *Cryptosporidium* spp. oocysts was examined using a modification of the Ziehl-Neelsen staining procedure. Bivariate and multivariate analyses were used to assess the association of *Cryptosporidium* infection and the general characteristics of the animals studied.

**Results:**

Overall, 112 (70 %, 95 % CI: 62.3–76.9) of the 160 small ruminants sampled were infected with *Cryptosporidium* spp. The prevalence of *Cryptosporidium* spp. infection in goats was 72.5 % (95 % CI: 61.4–81.9) and in sheep 67.5 % (95 % CI: 56.1–77.6). Small ruminants aged 1 month old had the highest (88.2 %; 95 % CI: 63.6–98.5) prevalence of infection. Prevalence varied from 60 % to 85 % among herds. Animal species, age, sex, breed, farm, town or cohabitation with cattle did not influence the prevalence of *Cryptosporidium* infection.

**Conclusions:**

A high prevalence of infection with *Cryptosporidium* spp. was observed in small ruminants from the Perote municipality in Veracruz, Mexico. Infection was widely distributed among sheep and goats regardless of their age, breed or farm location. Further research is required to identify risk factors for, and to assess the veterinary public health significance of *Cryptosporidium* infection among sheep and goats in the Mexican state of Veracruz.

## Background

*Cryptosporidium* is a zoonotic protozoan parasite of the Apicomplexa phylum and the Cryptosporidiidae family [1]. *Cryptosporidium* is an important pathogen in cattle and humans [2], and infection with this parasite in cattle may lead to economic losses due to morbidity and mortality [3, 4]. *Cryptosporidium* spp. infecting sheep and goats have been found to be of public health significance [5, 6].

Cryptosporidiosis, the disease caused by *Cryptosporidium*, is characterized by diarrhea, dehydration, and weight loss [7–9]. *Cryptosporidium* affects epithelial cells of the small intestine and occasionally stomach, gall bladder, liver, trachea, and lungs in a number of mammals including humans [10, 11]. *Cryptosporidium* is a widely distributed parasite and can infect more than 170 species of vertebrates [1]. Transmission of *Cryptosporidium* occurs mainly by the fecal-oral route [7]. In sheep and goats, cryptosporidiosis is an important enteric disease resulting in diarrhea and inefficient weight gains [12], and is more severe in young than in adult animals [13, 14]. Animals infected with *Cryptosporidium* shed a large (10^8^–10^9^ /g) number of oocysts [15]. These oocysts are source of infection for animals and humans [16]. In humans, infection with *Cryptosporidium* causes diarrheal disease [17], and chronic and fatal disease in immunocompromised individuals [18]. Additionally, *Cryptosporidium* infection has been linked to cancer in humans [4, 19].

Infection with *Cryptosporidium* in young sheep and goats may lead to morbidity and even mortality due to diarrhea [20–22]. Effective disease prevention requires an understanding of the environmental factors predisposing animal and human populations to infectious causes of diarrhea like *Cryptosporidium* spp. [23, 24]. However, very little is known about the infection with *Cryptosporidium spp*. in sheep and goats in Mexico in general and there is a lack of information about this infection in the southern Mexican state of Veracruz in particular. Therefore, we sought to determine the prevalence of infection with *Cryptosporidium* spp. in sheep and goats in the municipality of Perote in Veracruz State, Mexico.

## Methods

### Study design

This cross-sectional study was performed in the municipality of Perote in Veracruz, Mexico from July to December 2014. Perote, the capital of the municipality with the same name, is located in the central-west region of Veracruz State (19°34’N 97°15’W) at an altitude of 2400 meters above sea level and has a cold and dry climate with a mean annual temperature of 12 °C, and a mean annual rainfall of 493.6 mm.

### Sheep and goats studied

Eighty sheep and 80 goats from the Perote municipality in Veracruz State were included in the study. Convenience sampling was applied. The small ruminant population sampled was raised in eight farms where sheep and goats herds were kept. Farms were located in four towns: 1) Perote; 2) San Antonio Limón; 3) Los Molinos; and 4) La Gloria. Ten sheep and 10 goats were sampled from each farm. Sheep and goats were young (up to 3 months old). A fecal sample from the rectum was obtained from each animal. Fecal samples were placed in a plastic bag and analyzed a day after sampling. General data of the agroecosystem and animals studied were obtained with the aid of a questionnaire. Animal data included species, age, sex, breed, presence of diarrhea, and history of deworming. Agroecosystem data collected included farm location, nearest town, sharing water with other animals, and cohabitation with dogs or cattle.

### Laboratory tests

Small ruminant fecal samples were analyzed using a modification of the centrifuge-flotation method of Faust [25]. Briefly, three grams of fecal sample were homogenized in distilled water and centrifuged at 1500 rpm for 1 min. Then supernatant was removed and the homogenization and centrifugation processes were repeated. After removing the supernatant, the sediment was resuspended in ZnSO_4_ (44 %) and centrifuged. Finally, the supernatant was removed and sediment was examined. *Cryptosporidium* oocysts were detected by a modification of the Ziehl-Neelsen staining method [26]. Samples were examined under oil immersion objective (100x) and oocysts were identified based on morphological features.

### Statistical analysis

Results were analyzed with the aid of the software Microsoft Excel, SPSS version 15.0 (SPSS Inc. Chicago, Illinois) and STATA version 11.0. Descriptive statistics were used to determine the prevalence of *Cryptosporidium* infection in sheep and goats. We used the Pearson’s chi-squared test for comparison of infection frequencies among groups. The association between *Cryptosporidium* infection, animal characteristics, and agroecosystem features was assessed by bivariate and multivariate analyses. The dependent variable was positivity to *Cryptosporidium* by Ziehl-Neelsen staining per individual animal. Independent variables included in the multivariable analysis were those with a *P* value ≤0.10 in the bivariate analysis: age, town, and farm. Odds ratio (OR) and 95 % confidence interval (CI) were calculated and statistical significance was set at a *P* value of < 0.05.

### Ethics statement

The Bioethics and Animal Welfare Commission of the Facultad de Medicina Veterinaria y Zootecnia of Universidad Veracruzana approved this project. Owners of the sheep and goats gave their consent to study their animals. Adequate veterinary care was provided to all animals studied.

## Results and discussion

Infection with *Cryptosporidium* was found in 112 (70.0 %; 95 % CI: 62.3–77.0 %) of the 160 small ruminants studied. Of the 80 sheep examined, 54 (67.5 %; 95 % CI: 56.1–77.6) were positive for *Cryptosporidium* infection. Whereas of the 80 goats studied, 58 (72.5 %; 95 % CI 61.4–81.9 %) were positive for *Cryptosporidium* infection. These results provide for the first time evidence of *Cryptosporidium spp.* infection in sheep and goats in the municipality of Perote in Veracruz, Mexico.

Information about the prevalence of infection with *Cryptosporidium* in animals in Mexico is scant. The prevalence of *Cryptosporidium* infection in sheep reported here is higher than the 34.3 % prevalence of *Cryptosporidium* infection in sheep in northern Mexico [27]. We are not aware of reports about the prevalence of *Cryptosporidium* infection in goats in Mexico. Therefore, we cannot compare our results with those in other region in the country. In an international context, the prevalence of *Cryptosporidium* infection found in sheep in Veracruz is higher than the prevalences found in sheep in Spain (31–59 %) [28, 29], Poland (10.1 %) [30], Egypt (2.5 %) [31], Iran (11.3 %) [32], Iraq (13.3 %) [33], Tunisia (11.2 %) [34], India (1.8 %) [35], and Papua New Guinea (2.2 %) [36]. Similarly, the prevalence of *Cryptosporidium* infection found in goats in Veracruz is higher than prevalences found in goats in Spain (30 %) [29], Poland (0 %) [30], Iraq (17.7 %) [33], and Papua New Guinea (4.4 %) [36]. However, comparison of prevalences of *Cryptosporidium* infection in animals among countries should be interpreted with care because matching for characteristics of animals and their raising conditions is challenging.

Sheep and goats may serve as reservoirs of *Cryptosporidium* for human infections [36]. Outbreaks of infection with *Cryptosporidium* in schoolchildren associated with contact with lamb/goat kids have been reported [37]. The occurrence of these outbreaks 3 years apart, with the same parasite in the same farm suggested that *Cryptosporidium* might establish in the environment [37]. In addition, cryptosporidiosis has been associated with consumption of unpasteurized goat milk in children [38]. Therefore, our results should alert for the risk of infection in humans in Veracruz through contact with infected sheep and goats, and consumption of unpasteurized milk from infected goats. *Cryptosporidium* infection has been reported in animals other than sheep and goat in Veracruz, Mexico. Prevalence of *Cryptosporidium* infection in cattle in several municipalities in Veracruz varied from 14.3 % to 75 % [39, 40]. Sheep, goats, and cattle are frequently raised together in Veracruz, and this interaction along with poor hygienic sanitary conditions in the agroecosystems where they are raised might account for transmission of *Cryptosporidium* infection among animal species [41]. A number of *Cryptosporidium* species/genotypes infecting sheep has been described including *C. parvum*, *C. bovis*, *C. cervine* [42–44], and *C. hominis* [42]. Whereas some *Cryptosporidium* species/genotypes infecting goats include *C. xiaoi* [36, 45], *C. parvum*, and *C. hominis* [36].

We sought to determine the association between the general characteristics of animals, their environment, and *Cryptosporidium* infection. The prevalence of *Cryptosporidium* infection did not vary significantly with small ruminant species, age, sex, or breed of the animals studied. Neonatal diarrhea in small ruminants is one of the most frequent syndromes during the first week of age and leads to important economic losses due to mortality and delayed growth. *Cryptosporidium* is a well-known cause of neonatal diarrhea in sheep [46]. However, in our study the history of diarrhea in the animals studied was not associated with *Cryptosporidium* infection. This finding agrees with that found in a study of lambs and adult sheep in Poland [30]. In the present study, the effect of dogs on *Cryptosporidium* prevalence could not be determined because all farms sampled had dogs present. We found no association between *Cryptosporidium* prevalence and a history of deworming or cohabitation with cattle. All sheep and goats studied shared water with other animals of the same farms. Table 1 shows a correlation between *Cryptosporidium* infection and general characteristics of sheep and goats and the agroecosystem where they were raised. Bivariate analysis of these characteristics showed three variables with a *P* < 0.10: age, towns and farms. Multivariate analysis of the three variables with a *P* < 0.10 (age, towns and farms) obtained in bivariate analysis showed no association with *Cryptosporidium* infection. The failure to find an association between the presence of infection and characteristics of the animals may have been due to the small sample size. Further studies with a larger sample size to determine the risk factors associated with *Cryptosporidium* infection in sheep and goats should be conducted to assess the veterinary public health burden of cryptosporidiosis in Mexico. In addition, further research with molecular methods to determine the *Cryptosporidium* genotypes, and subtyping of *C. parvum* or *C. ubiquitum* at the gp60 locus [47, 48] in sheep and goats in Mexico are needed.

**Table 1:** Correlation of *Cryptosporidium* infection with the general characteristics of the animals(sheep and goats together) and the agroecosystem where they were raised

	No. of animals	*Cryptosporidium* infection			95 % Confidence	*P*
Characteristic	studied	No.	%	OR	interval	value
Species						
Sheep	80	54	67.5	1.00		
Goats	80	58	72.5	1.26	0.64–2.50	0.49
Age (months)						
< 1	17	15	88.2	3.50	0.77–16.37	0.08
1	124	84	67.7	1.00		
2	19	13	68.4	1.03	0.36–2.91	0.95
Sex						
Male	89	58	65.2	1.00		
Female	71	54	76.1	1.69	0.84–3.41	0.13
Breed of Sheep						
Pelibuey	45	30	66.7	1.33	0.39–4.44	0.63
Dorper	20	15	75	2.00	0.47–8.49	0.34
Kathadin	15	9	60	1.00		
Breed of goats						
Mixed	46	32	69.6	1.00		
Nubian	34	26	76.5	1.42	0.51–3.90	0.49
Diarrhea						
Yes	52	33	63.5	1.00		
No	108	79	73.1	1.56	0.77–3.18	0.21
Deworming						
Yes	80	56	70	1.00		
No	80	56	70	1.00	0.50–1.96	1
Town						
Perote	40	27	67.5	1.24	0.49–3.12	0.63
San Antonio Limón	40	32	80	2.40	0.87–6.55	0.08
Los Molinos	40	28	70	1.40	0.55–3.55	0.47
La Gloria	40	25	62.5	1.00		
Farm						
1	20	14	70	1.55	0.41–5.76	0.5
2	20	13	65	1.23	0.34–4.46	0.74
3	20	15	75	2.00	0.51–7.72	0.31
4	20	17	85	3.77	0.82–17.25	0.07
5	20	14	70	1.55	0.41–5.76	0.5
6	20	14	70	1.55	0.41–5.76	0.5
7	20	12	60	1.00		
8	20	13	65	1.23	0.34–4.46	0.74
Cohabitation with cattle						
Yes	100	71	71	1.13	0.56–2.27	0.72
No	60	41	68.3	1.00		

## Conclusions

The prevalence of infection with *Cryptosporidium spp* among sheep and goats in the Perote municipality in Veracruz, Mexico is relatively high. Infection was widely distributed among small ruminants regardless of their age, breed or the agroecosystem where they were raised. Further research to identify risk factors for *Cryptosporidium* infection in sheep and goats and the zoonotic potential of cryptosporidiosis in Veracruz State is needed.
